# Attainment of Sexual Maturity and Gonadotropin Priming in Gilts Determine Follicular Development, Endocrine Milieu and Response to Ovulatory Triggers

**DOI:** 10.3390/ijms23169190

**Published:** 2022-08-16

**Authors:** Pawel Likszo, Katarzyna Gromadzka-Hliwa, Jan Klos, Monika M. Kaczmarek, Adam J. Ziecik

**Affiliations:** 1 Department of Hormonal Action Mechanisms, Institute of Animal Reproduction and Food Research, Polish Academy of Sciences, 10-748 Olsztyn, Poland; 2 Molecular Biology Laboratory, Institute of Animal Reproduction and Food Research, Polish Academy of Sciences, 10-748 Olsztyn, Poland

**Keywords:** preovulatory follicles, gonadotropins, puberty, steroidogenesis, pig

## Abstract

The routine procedure of estrous cycle synchronization in pigs allows for the use of gonadotropins to stimulate ovarian activity. The applied protocols of eCG and hFSH priming similarly affected development of ovarian follicles in two classes 3–6 mm and >6 mm of diameter, however, the number of small follicles (<3 mm) was 2-fold higher in hFSH- than in eCG-primed prepubertal gilts. The attainment of sexual maturity increased concentration of estradiol, testosterone and androstenedione in the follicular fluid of hFSH/eCG-primed gilts, however, prostaglandin E2 and F2α metabolite increased in mature hFSH- and eCG-primed gilts, respectively. The maturity increased mRNA and/or protein expression of key steroidogenic enzymes, prostaglandin synthases or luteinizing hormone receptors in follicular walls. Both hormonal primers played a moderate role in affecting expression of steroidogenic enzymes in follicular walls. In vitro studies showed higher estradiol production in r-hLH (*p* = 0.04)- and r-hCG (*p* = 0.049)-stimulated follicular walls of mature gilts than in prepubertal hFSH-primed gilts. Both ovulatory triggers decreased the abundance of LHCG/FSH mRNA receptors in follicular walls, which mimic downregulation of these receptors by a preovulatory LH surge, confirmed in vivo. These data revealed the importance of sexual maturity in the protection of the estrogenic environment, and the selective, moderate role of eCG and FSH in the activation of steroidogenic enzymes in preovulatory follicles.

## 1. Introduction

The natural way to control the estrous cycles in weaned sows and postpubertal gilts is through the observation of estrous behavior [1]. However, due to variability in the interval between the onset of estrus and the course of preovulatory luteinizing hormone (LH) surge [2], and the actual time of ovulation [3], hormonal treatments are used in different protocols to control the reproductive function of sows and gilts [4].

The routine synchronization procedure usually consists of three distinct phases: (1) quiescence of the hypothalamic-pituitary-ovarian axis and inhibition of follicular development [5,6], (2) administration of equine chronic gonadotropin (eCG) or follicle stimulating hormone (FSH) to enhance the development of preovulatory follicles [7,8] and (3) challenge of human chronic gonadotropin (hCG) or administration of gonadotropin-releasing hormone (GnRH) agonist (A) to stimulate the release of endogenous LH to start the ovulation process [9]. A combined administration of eCG and hCG was also used to exert a gonadotropin effect on developing follicles [10].

Altrenogest, a synthetic steroid with progestagenic activity, is used to synchronize the cycle of sows and gilts, as it postpones the onset of the follicular phase. The proper identification of mature gilts for altrenogest treatment can be challenging, since in some herds, only 1/3 of gilts ovulated in the first estrus, about 20 percent ovulated before the first behavioral estrus and over 40% did not ovulate during the first estrus [11]. The false detection of ovulation and too-early hormonal treatment may lead to the development of follicular cysts in prepubertal gilts. Our previous results indicated that altrenogest treatment, followed by eCG and hCG challenges caused a relatively high (66.6%) incidence of follicular cyst formation in prepubertal gilts. In contrast, in gilts that had already passed their first natural ovulation, the same treatment led to a reduction of follicular cyst appearance (14%) [12].

To explain the possible mechanism of the combined action of altrenogest and exogenous gonadotropins in follicular development and their potential transformation into cysts, we focused our recent studies on the effect of altrenogest [13] and exogenous gonadotropin (hCG vs. GnRH-A-induced LH) [14] administration on ovarian folliculogenesis, endocrine and molecular milieus of the preovulatory follicles in prepubertal and sexually mature gilts. We showed that altrenogest equivocally affects folliculogenesis and the development of growing follicles. In addition, different endocrine properties of exogenous hCG and endogenous LH with a potent progestational/androgenic role of hCG and estrogenic/pro-developmental function of LH were observed.

Other than the explanation of applied exogenous agents’ (altrenogest and hCG) role in the processes of estrus synchronization in gilts, there is still need for the elucidation of eCG’s participation in follicular development. Equine chorionic gonadotropin is produced in equine endometrial coups after invasion of uterine epithelium by fetal chorionic cells [15]. It is synthetized as a classic gonadotropin α and β chain heterodimer and is post-translationally glycosylated [16]. The primary structure of eCG is identical to equine LHβ [17]. A strong attachment of sialic acid imports to eCG is one of the most important properties of its long biological life [18]. When eCG was injected into sheep [18] and cattle [19], they displayed an elimination half-life in blood of 21 and 45 h, respectively. The half-life of eCG in gilts after intravenous injection is about 15 h [20]. In mares, eCG binds to LH receptors, but it exerts FSH and LH activity in non-equine species by stimulation of both FSH and LH receptors [21]. It is thought that eCG expresses predominantly higher FSH than LH activity in the female pig [22], though precise studies have never been performed.

The most widespread use of eCG is strictly connected to its FSH activity applied to induce estrus in immature animals, including pigs [23]. Equine chorionic gonadotropin is frequently used for estrus synchronization in gilts and post-weaned sows alone [6] or in combination with hCG [10]. However, the dual FSH and LH activity of eCG and its long circulation life after administration can be a reason for the high percentage of ovarian cyst appearances in the pig [24,25,26] and other domestic animals [27]. Early studies by Flowers and co-workers [28] showed that eCG may stimulate follicular development, estradiol production and ovulation without increasing the secretion of FSH in prepubertal gilts.

Despite its widespread use, the manner in which eCG stimulates ovarian activity is not completely understood. Thus, the objectives of the present research were to: (1) compare the effect of eCG and hFSH on the development of follicles in altrenogest-primed prepubertal and mature gilts, (2) show whether eCG and hFSH challenge can differentially affect an endocrine and molecular pattern of ovarian follicles in vivo and (3) compare the responsiveness of the follicular walls (granulosa and theca layers) of eCG/FSH-primed gilts to hLH and hCG stimulation in vitro.

## 2. Results

### 2.1. The Sexual Maturity Status Affects the Number of Small Visible Ovarian Follicles in hFSH- and eCG-Primed Gilts

**Figure 1:**
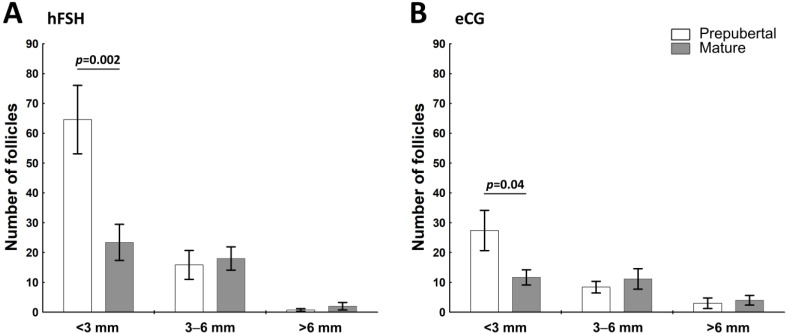
Number of ovarian follicles differs among pre-pubertal or mature gilts after hormonal treatment (hFSH—(**A**); eCG—(**B**)) in gilts. Number of ovarian follicles in three different size categories (<3, 3–6, and >6 mm in diameter) is shown for prepubertal and mature gilts. Data are presented as mean ± SEM (n = 6 per group). Data were analyzed using Student’s *t*-test in each follicle category. Line with a *p*-value denote significant differences between prepubertal and mature gilts.

The number of visible follicles on both left and right ovaries in three different size categories (small—<3; medium—3–6; and large—>6 mm in diameter) is shown in Figure 1.

The hFSH- and eCG-primed prepubertal gilts had 2.5–2.8 more small follicles (<3 mm; *p* = 0.002 and *p* = 0.04) than mature gilts. However, the absolute number of small follicles was 2.4- fold higher in hFSH- than eCG-primed gilts (64.57 ± 11.47 vs. 27.4 ± 6.76; *p* < 0.05). In hFSH- and eCG-primed gilts, the number of medium and large follicles did not differ between prepubertal and mature gilts.

### 2.2. The Sexual Maturity Status Affects the Hormonal Milieu in Ovarian Follicles of hFSH- and eCG-Primed Gilts

**Figure 2:**
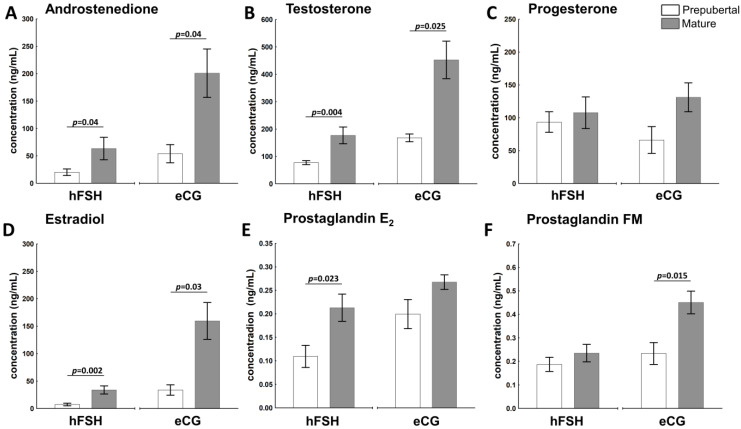
Hormonal milieu of the follicular fluid is affected by the sexual maturity (pre-puberty or maturity) of hFSH- and eCG-treated gilts. The follicular fluid concentration of A4 (**A**), T (**B**), P4 (**C**), E2 (**D**), PGE2, (**E**) and PGFM (**F**) is shown for prepubertal and mature gilts. Data are presented as mean ± SEM (n = 6 per group). Data were analyzed using Student’s *t*-test for each hormonal treatment. Line with a *p*-value denotes significant differences between prepubertal and mature gilts.

The attainment of sexual maturity affected androstenedione (A4), testosterone (T) and estradiol (E2) concentration in the follicular fluid of hFSH- and eCG-primed gilts (Figure 2A,B,D; respectively). The steroid hormone levels (A4, T and E2) were about 2-fold higher in mature vs. prepubertal hFSH-primed gilts (*p* = 0.04, *p* = 0.004 and *p* = 0.002; respectively), whereas in eCG-primed gilts 4-(*p* = 0.04), 3-(*p* = 0.025), and 6-fold (*p* = 0.03) increase was noticed, respectively. Gaining maturity also increased level of prostaglandin E2 (PGE2) in hFSH (*p* = 0.023)- and prostaglandin F2α metabolite (13,14-dihydro-15-keto- F2α; PGFM) in eCG (*p* = 0.015)-primed gilts (Figure 2E,F). Concentration of progesterone (P4) was maintained at almost constant levels in hFSH- and eCG-primed gilts, independent of the sexual maturity status (Figure 2C).

Correlations between the level of follicular E2 and T, A4 and P were high (r = 0.9583, r = 0.9155 and r = 0.8359; respectively; *p* = 0.0001) in hFSH-primed gilts. A positive correlation of E2 and T (r = 0.7715, *p* = 0.005), A4 (r = 0.6089, *p* = 0.047), but not P4 (r = 0.3021, *p* = 0.367) were observed in eCG-primed gilts. In addition, levels of T and A4 in follicular fluid also correlated with P4 in hFSH (r = 0.8155, *p* = 0.001 and r = 0.8886, *p* = 0.0001)- and in eCG (r = 0.6381, *p* = 0.035 and r = 0.6556, *p* = 0.029)-primed gilts, respectively. Concentration of P4 was negatively correlated with CYP19A1 protein abundance (r = −0.7855, *p* = 0.007).

### 2.3. The Sexual Maturity Lead to Molecular Changes in Follicular Walls of Ovarian Follicles of hFSH- and eCG-Primed Gilts

#### 2.3.1. Factors Related to Progesterone and Estrogen Synthesis

**Figure 3:**
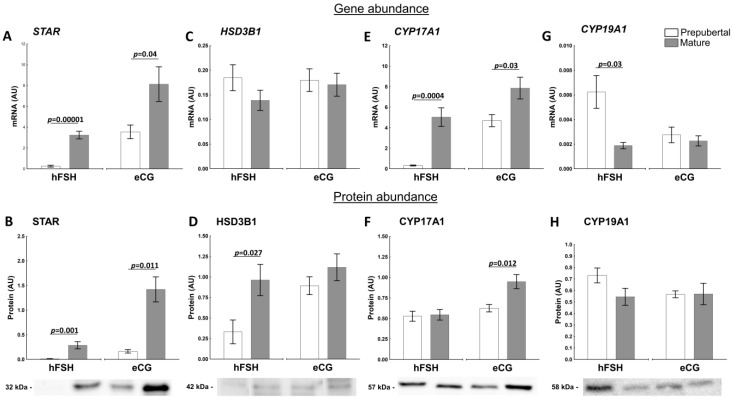
Sexual maturity status (pre-puberty or maturity) change abundance of factors related to production of androgens and estrogens in ovarian follicles of hFSH- and eCG-primed gilts. The expression of STAR (**A**,**B**), HSD3B1 (**C**,**D**), CYP17A1 (**E**,**F**) and CYP19A1 (**G**,**H**) in prepubertal and mature gilts was evaluated. Gene expression was normalized to the geometric mean of *ACTB* and *GAPDH* (AU), identified as the best reference genes by NormFinder algorithm. Protein levels were normalized to total protein content (AU) using TGX Stain-Free gel technology (**B**,**D**,**F**,**H**). Uncropped blots are presented in Supplementary Figure S1. Data were analyzed using Student’s *t*-test for each treatment and are presented as mean ± SEM (n = 6 per group). Line with a *p*-value denotes significant differences between prepubertal and mature gilts. AU—arbitrary units.

Steroidogenic acute regulatory protein (STAR) is a key factor in regulating the timing and rate of steroidogenesis by controlling the movement of cholesterol across mitochondrial membranes in the ovarian follicles [29]. The sexual maturity attainment significantly affected *STAR* mRNA (Figure 3A) and protein abundance (Figure 3B) in follicles in both groups of hFSH- and eCG-primed gilts. The STAR protein expression was higher by about 6-fold in mature compared with prepubertal (*p* = 0.001; Figure 3B) eCG-primed gilts.

The STAR protein correlated with E2 (r = 0.8885, *p* = 0.0001) and T (r = 0.6820, *p* = 0.03) in hFSH and with E2 (r = 0.9829, *p* = 0.0001), T (r = 0.7476, *p* = 0.008) and A4 (r = 0.7694, *p* = 0.009) in eCG-primed gilts.

Hydroxy-delta-5-steroid dehydrogenase 3 beta- and steroid delta-isomerase 1 (HSD3B1) regulates an essential step in the biosynthesis of steroid hormones (i.e., P4, A4 and T). Although mRNA levels of *HSD3B1* were maintained almost constant in our experimental setting (Figure 3C), its protein abundance was affected by the maturity (*p* = 0.027) in hFSH-primed gilts (Figure 3D).

There was a positive correlation between the HSD3B1 protein abundance and A4 concentration in follicular fluid in hFSH (r = 0.5767, *p* = 0.049)- and eCG (r = 0.7326, *p* = 0.016)-primed gilts. The positive correlation between HSD3B1 protein abundance and T level in follicular fluid in eCG-primed gilts was noticed (r = 0.6904, *p* = 0.04). In addition, HSD3B1 protein was positively correlated with FSHR protein abundance (r = 0.6939, *p* = 0.038) in hFSH-primed gilts.

Level of cytochrome P450 family 17, subfamily A member 1 (CYP17A1) mRNA an enzyme that occupies an important role in the pathways of porcine follicular steroidogenesis [1,22], was clearly depended on the maturity of hFSH- and eCG-primed gilts (*p* = 0.0004 and *p* = 0.03, respectively; Figure 3E). Although protein CYP17A1 abundance was not affected by maturity status in hFSH-primed gilts (Figure 3F), eCG priming increased protein levels in mature gilts (*p* = 0.012). At the tested stage of the follicular development, after hFSH or eCG priming, there was no correlation of the CYP17A1 protein with follicular hormones. However, there was a positive correlation between the CYP17A1 and CYP19A1 protein abundances in hFSH (r = 0.8720, *p* = 0.001)- and eCG (r = 0.7581, *p* = 0.007)-primed gilts. Additionally, the protein abundance of CYP17A1 was correlated with FSHR (r = 0.7209, *p* = 0.019) in eCG-primed gilts.

Cytochrome P450 family 19, subfamily a member 1 (CYP19A1) is a key factor in estrogen biosynthesis [30]. The abundance of *CYP19A1* mRNA in hFSH-primed gilts was about 3-fold higher in prepubertal than mature gilts (Figure 3G). In addition, the CYP19A1 protein level was negatively correlated with follicular P4 (r = −0.7855, *p* = 0.007).

**Figure 4:**
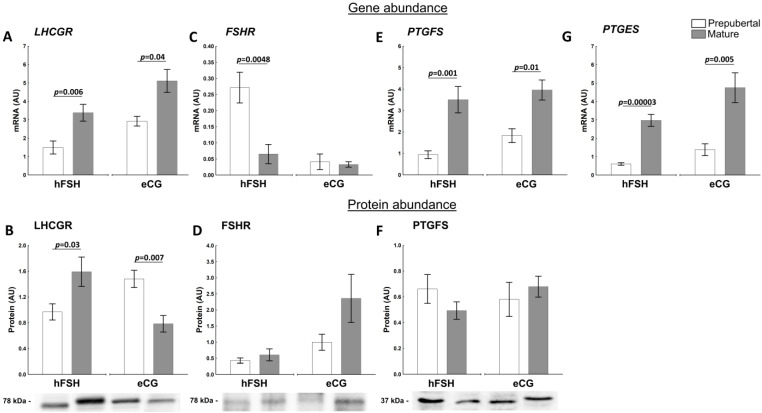
Sexual maturity status (pre-puberty or maturity) affect abundance of factors related tosteroid hormone synthesis and gamete production in ovarian follicles of hFSH- and eCG-primed gilts. The abundance of LHCGR (**A**,**B**), FSHR (**C**,**D**), PTGFS (**E**,**F**) and *PTGES* (**G**) in prepubertal and mature gilts was evaluated. Gene expression was normalized to the geometric mean of *ACTB* and *GAPDH*, identified as the best reference genes by NormFinder algorithm. Protein levels were normalized to total protein content using TGX Stain-Free gel technology (**B**,**D**,**F**). Uncropped blots are presented in Supplementary Figure S2. Data were analyzed using Student’s *t*-test for each treatment and are presented as mean ± SEM (n = 6 per group). Line with a *p*-value denotes significant differences between prepubertal and mature gilts. AU—arbitrary units.

LHCGR and FSHR play an essential role in the regulation of ovarian steroidogenesis, and their activity are under the control of the hypothalamic-pituitary-gonadal axis [1]. The sexual maturity affected *LHCGR* mRNA abundance (Figure 4A) in the follicles of hFSH- and eCG-primed gilts (*p* = 0.006 and *p* = 0.04, respectively). Similarly, higher protein abundance of LHCGR was found in mature hFSH-primed gilts (*p* = 0.03). Interestingly, LHCGR protein abundance was lower (*p* = 0.007) in follicles of mature than of prepubertal eCG-primed gilts (Figure 4B). The LHCGR protein level was positively correlated with FSHR protein abundance (r = 0.8282, *p* = 0.011).

*FSHR* mRNA abundance in follicular walls was the highest in prepubertal hFSH-primed gilts (*p* = 0.0048 vs. mature; Figure 4C). A western blot analysis of FSHR protein did not show differences in abundance between prepubertal and mature hFSH- and eCG-primed gilts (Figure 4D).

#### 2.3.2. Factors Related to the Production and Action of PGs

Prostaglandins regulate the ovarian cycle. Specifically, PGF2α and PGE2 concentrations increase during follicular maturation [31] and induce follicular rupture [32]. Sexual maturity significantly affected the mRNA abundance of both PGF2α (PTGFS) and PGE2 (PTGES) synthases in ovarian follicles. *PTGFS* mRNA abundance was higher in mature than in prepubertal hFSH (*p* = 0.001)- and eCG (*p* = 0.01; Figure 4E)-primed gilts, similar to *PTGES* mRNA (*p* < 0.00003, *p* = 0.005, respectively; Figure 4G). Protein levels of PTGFS were maintained at almost constant levels in our experimental setting (Figure 4F). Unfortunately, the protein abundance for PTGES could not be measured due to the unavailability of the specific porcine antibodies.

### 2.4. Effect of r-hLH and r-hCG In Vitro Stimulation on Molecular Changes in Follicular Walls of Prepubertal and Mature hFSH- and eCG-Primed Gilts

To study the follicular wall’s (granulosa and theca) responsiveness to ovulatory stimuli, we used human recombinant (r-LH and r-hCG) preparations to avoid any potential contamination seen in natural standards. In the porcine granulosa cells, ED50 doses of r-hLH and r-hCG were about 2-fold more potent in the stimulation of E2 production than native pLH [13]. ED50 doses of r-hLH and r-hCG were about 4/9 and about 1.5/2 more potent than pLH in stimulation of E2/P4 production in follicular wall explants in vitro, respectively (Supplementary Figure S3A,B). Both recombinant gonadotropins showed a dose-dependent effect on the production of E2 and P4 (Supplementary Figure S3C–F).

#### 2.4.1. E2 and P4 Production

**Figure 5:**
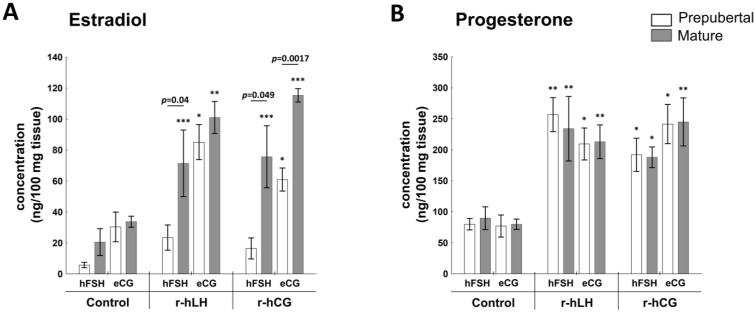
Hormonal response of the follicular walls to r-hLH and r-hCG treatment differs in pre-pubertal and mature gilts primed in vivo with hFSH or eCG. Secretion of E2 (**A**) and P4 (**B**) by r-hLH- and r-hCG-in vitro-stimulated follicular wall explants is shown for prepubertal and mature gilts primed in vivo with hFSH or eCG. Data were analyzed using one-way ANOVA with Tukey post-hoc tests and are presented as mean ± SEM (n = 6 per group); * p < 0.05; ** p < 0.001; *** p < 0.0001 for control vs. r-hLH or r-hCG treatment. Line with a *p*-value denotes significant differences between prepubertal and mature gilts.

There were no differences in E2 concentration in the control culture medium after the incubation of follicular wall explants without hLH or hCG (Figure 5A). Both triggers (r-hLH and r-hCG) increased E2 secretion in follicular wall explants collected from prepubertal eCG- and mature FSH- and eCG-primed gilts compared to control (*p* < 0.05), but not prepubertal hFSH-challenged gilts. The attainment of sexual maturity increased E2 production by 3-fold (*p* = 0.04) in r-hLH- and 5-fold (*p* = 0.049) in r-hCG-treated explants collected from hFSH-primed gilts. Such a difference was not observed in the follicular walls of eCG-primed gilts exposed to r-hLH. However, r-hCG in vitro treatment enhanced E2 production by 2-fold in explants collected from mature vs. prepubertal eCG-primed gilts (*p* = 0.0017; Figure 5A).

The concentration of P4 after in vitro stimulation of follicular explants with r-hLH or r-hCG was not effected by either maturity or hFSH/eCG-priming. Both r-hLH and r-hCG triggers similarly increased (about 2.5-fold) production of P4 in the follicular explants collected from hFSH- and eCG-primed prepubertal and mature gilts compared with control (*p* < 0.05; Figure 5B).

#### 2.4.2. Receptors Related to Progesterone and Estrogen Synthesis

**Figure 6:**
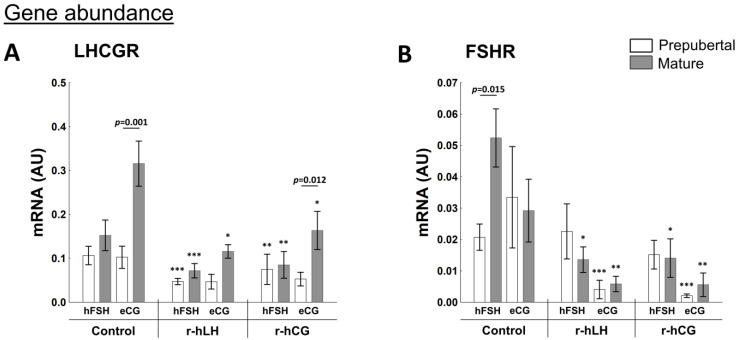
r-hLH and r-hCG in vitro treatments affect abundance of receptors related to progesterone and estrogen synthesis in ovarian follicles of prepubertal and mature gilts primed in vivo with hFSH or eCG. Evaluation of mRNA levels was performed for *LHCGR* (**A**) and *FSHR* mRNA (**B**) after exposition of follicular walls to r-hLH and r-hCG. Gene expression was normalized to the geometric mean of *ACTB* and *GAPDH*, identified as the best reference genes by NormFinder algorithm. Data were analyzed using one-way ANOVA with Tukey post-hoc tests and are presented as mean ± SEM (n = 6 per group); * p < 0.05; ** p < 0.001; *** p < 0.0001 for control vs. hLH or hCG treatment. Line with a *p*-value denotes significant differences between prepubertal and mature gilts. AU—arbitrary units.

An increased *LHCGR* mRNA abundance in control explants collected from eCG-primed mature vs. prepubertal gilts (*p* = 0.001) was indicated. A lower *LHCGR* mRNA abundance was observed in explants collected from prepubertal vs. mature eCG-primed gilts after in vitro delivery of the ovulatory stimuli—r-hCG (*p* = 0.012). In general, both in vitro treatments (r-hLH and r-hCG) decreased the mRNA abundance of *LHCGR* in follicular explants of prepubertal and mature hFSH- or eCG-primed gilts and reached the significance when compared to control (*p* < 0.05; Figure 6A).

In control, the mean abundance of *FSHR* mRNA was 2.5-fold higher in hFSH-primed mature vs. prepubertal (*p* = 0.015) gilts (Figure 6B). Moreover, decreased *FSHR* mRNA abundance after r-hLH and r-hCG treatment of follicular walls collected from eCG- and hFSH-primed gilts (*p* < 0.05) was found compared to control, except for the explants collected from prepubertal hFSH-primed gilts.

### 2.5. Hormonal Milieu and LHCGR and FSHR Levels in Follicular Walls Change before Ovulation

**Figure 7:**
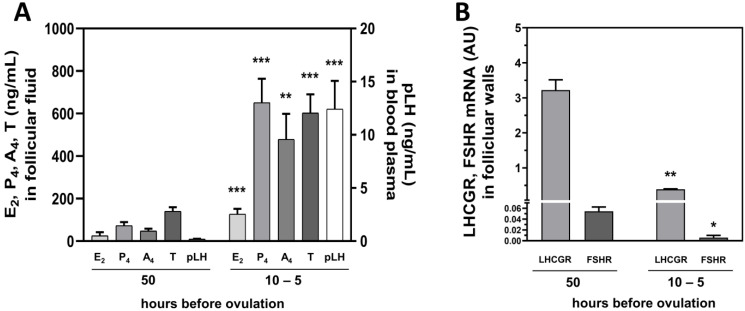
Hormonal milieu and dynamics of LHCGR and FSHR levels in follicular walls before ovulation. Concentrations of E2, P4, A4 and T in follicular fluid and pLH in blood plasma 50 and 10 to 5 h before ovulation (**A**). Expression of *LHCGR* and *FSHR* mRNA in preovulatory follicles walls 50 and 10 to 5 h before ovulation (**B**). * p < 0.05; ** p < 0.001; *** p < 0.0001 for hormones/receptors at 50 h vs. 10-5 h before ovulation (Student’s *t*-test). AU—arbitrary units.

The concentrations of E2, P4, A4 and T in follicular fluid and porcine LH (pLH) concentration in blood plasma were higher at 10 to 5 than 50 h before ovulation (*p* < 0.001; Figure 7A). Whereas expression of *LHCGR* and *FSHR* mRNA in the walls of the preovulatory follicles was about 6-fold lower at 10 to 5 than 50 h before ovulation (*p* < 0.001 and *p* < 0.05, respectively; Figure 7B). There was a positive correlation between the level of blood plasma pLH and follicular fluid E2 (r = 0.4463, *p* = 0.043), P4 (r = 0.7961, *p* < 0.001), A4 (r = 0.9097, *p* < 0.001) and T (r = 0.7601, *p* < 0.001). The level of blood plasma pLH were also a negatively correlated with *LHCGR* and *FSHR* mRNA abundance in preovulatory follicle walls (r = −0.7761, *p* = 0.005 and r = −0.3869, *p* = 0.011, respectively).

## 3. Discussion

This work represents the first in-depth analysis of porcine follicle responses to in vivo hormonal priming with eCG and hFSH in altrenogest-primed prepubertal and mature gilts, tested further upon in vitro delivery of ovulatory triggers—LH and hCG. Using multilevel investigation, we were able to assess the effect of eCG and hFSH priming on the development of follicles and their endocrine and molecular milieus. Further in vitro studies allowed us to investigate the responsiveness of the follicular walls of eCG- or FSH-primed prepubertal or mature gilts to LH and hCG in vitro stimulation.

A number of visible ovarian follicles in the altrenogest- and then eCG- or hFSH-primed prepubertal and mature gilts revealed the effect of maturity status and hormonal priming on follicular development. The highest number of small (<3 mm) follicles was found in prepubertal hFSH-primed gilts. Altrenogest priming and specific properties of FSH can be the main reason for such an outcome. We showed that altrenogest increase the number of total antral follicles as well as decrease the percentage of atretic small antral follicles. Additionally, it increased the percentage of healthy antral follicles [13]. On the other hand, small follicles predominantly bind FSH and show maximal *FSHR* gene expression [31,33]. Injection of FSH led to growth promotion of small follicles in gilts [7,8,34]. It was shown that FSH inhibits granulosa cell apoptosis and can rescue granulosa cells from atresia and apoptosis in mice [35], rats [36] and pigs [37,38,39]. In the presented work, the highest *FSHR* mRNA levels occurred in preovulatory follicles collected from prepubertal hFSH-primed gilts, however, the abundance of FSHR mRNA and protein were not measured in small follicles.

The pattern of A4, T and E2 secretion in preovulatory follicles was similar in experimental groups. The concentration of these steroid hormones in follicular fluid was relatively low in prepubertal hFSH- and eCG-primed gilts, but eCG visibly potentiated E2 levels both in prepubertal and mature gilts. Androgens and estradiol concentrations in follicular fluid followed the expression patterns of mRNA and protein for STAR, HSD3B1 and CYP17A1, as well as protein for FSHR. STAR and CYP17A1 control the rate-limiting steps of de novo steroidogenesis. STAR provides cholesterol entry into the mitochondria and CYP17A1 guarantees conversion of pregnenolone towards androstenedione and testosterone. Interestingly, mRNA abundance of aromatase (*CYP19A1*), expressed by both granulosa and theca layers [30,40], was the highest in the follicular walls of hFSH-primed prepubertal gilts compared with counterpart prepubertal eCG-primed and both mature groups. However, the expression of *CYP19A1* mRNA did not correlate with this enzyme protein abundance and E2 concentration in the follicular fluid. Interestingly, the level of E2 in follicular fluid did not follow the pattern of CYP19A1 protein abundance, but rather STAR, CYP17A1 and FSHR proteins. It is thought that CYP17A1, the last enzyme in the biosynthesis of androgens, becomes the rate-limiting enzyme of follicular estrogen synthesis in pigs [22]. Furthermore, our recent results and previous studies [13,14] confirmed the hypothesis that the availability of substrate, but not the magnitude of *CYP19A1* mRNA/protein expression, is critical for maintaining E2 synthesis in the porcine preovulatory follicle.

Gene expression of the *HSD3B1* enzyme involved in P4 production by converting pregnenolone to progesterone was not affected by maturity in both hFSH- and eCG-primed gilts. The protein abundance of HSD3B1 did not differ between the particular groups of prepubertal and mature eCG-primed gilts. It could partially explain, why P4 concentrations in the follicular fluid did not differ between experimental groups. The relatively low levels of P4 in the follicular fluid could be caused by the inhibitory effect of altrenogest pretreatment on the production of progesterone in both prepubertal and mature gilts [13]. At the tested stage of the follicular development, after hFSH or eCG priming, there was no correlation of *LHCGR* mRNA and protein with the follicular P4 or E2 levels. This could reflect the different pattern of LHCGR expression in granulosa cells and theca layer during the follicle maturation [1]. Our recent results showed that the administration of ovulation stimuli in vivo, i.e., hCG and GnRH-A (to induce a native LH release), affected steroidogenesis in preovulatory follicles, and that the attainment of sexual maturity was strongly visible in altrenogest- and eCG-primed gilts [14]. In this study, hCG preferentially affected production of P4, A4 and T, however, native LH and sexual maturity act as an advantage for E2 concentration in the follicular fluid.

The study performed in vitro using the follicular wall explants showed the significant stimulatory effect of sexual maturity and gonadotropin priming on the in vitro r-hLH- or r-hCG-stimulated production of E2, except for prepubertal hFSH-primed gilts, as both triggers were not able to induce E2 production. This can be explained by the low LH receptor mRNA and protein concentration in follicular walls of prepubertal hFSH-primed gilts. In contrast, prepubertal gilts primed with eCG expressed higher LH receptor protein levels that could aid E2 production (see Figure 5B).

The overnight incubation of the follicular tissues with ovulations triggers, i.e., r-hLH and r-hCG, also caused a significant decrease of *LHCGR* and *FSHR* mRNA abundance. Since these results could refer to an important regulation of hormone synthesis in the preovulatory follicle, we determined the steroid hormone concentrations in follicular fluid and LH blood levels in gilts approximately 50 or 10 to 5 h before ovulation. In addition, *LHCGR* and *FSHR* mRNA abundance was tested in follicular walls at the same time points (Experiment 3). Interestingly, the concentration of steroid hormones in follicular fluid and of LH in blood were negatively correlated with both gonadotropin receptors in follicular walls just before ovulation. Apparently, the results of in vitro treatment of follicular walls with r-hLH or r-hCG mimic the in vivo hormonal status occurring during the preovulatory LH surge that causes down regulation of both LHCGR and FSHR.

In conclusion, this study, together with our two already published studies [13,14], indicates that sexual maturity, altrenogest and exogenous gonadotropins significantly affect endocrine and molecular regulators of ovarian follicle development and function in gilts. Whereas the diversity of hCG and LH (released by delivered GnRH) action was presented in detail by Ziecik and co-workers (2021), this study compared eCG- and hFSH-priming effects in gilts. The main finding of this study is the quite similar follicular developmental and steroid hormone secretion pattern after priming gilts with hFSH or eCG, however, these were dependent on the attainment of sexual maturity. Both exogenous gonadotropins played moderate roles in the regulation of mRNA/protein abundance of some steroidogenic enzymes and FSH/LH receptors in follicular walls. An in vitro study revealed similar effectiveness for both ovulation stimuli (hLH/hCG) in the production of steroid hormones and down regulation of LH receptors in follicular walls, confirming the same regulatory mechanisms acting in in vitro and in vivo conditions. Because the administration of exogenous gonadotropins creates favorable endocrine conditions for the development of ovarian cysts, more attention should be given to the wise selection of hormonal stimulation protocols at farms, including exogenous gonadotropins cured and the sexual status of gilts.

## 4. Materials and Methods

### 4.1. Selection of Animals and Experimental Groups Recruitment

#### 4.1.1. Experiment 1 [In Vivo]

A total of twenty-four crossbred gilts of similar age—165-day old, and weight—115 kg, from one commercial herd were used in this experiment. Crossbred gilts were contacted with a mature boar every day for 14 days and then, at approximately 180 days of age, were used in two trials to create experimental groups, as previously described [13]. Briefly, gilts considered as being in the first natural estrus formed a future sexually mature (M) pool of gilts, which were recruited at 185–195 days of age. A set of gilts without estrus symptoms at 180 days of age was designed to form prepubertal (P) groups. Both groups (P and M) were fed 20 mg daily of altrenogest (Suifertil, Medica, Poland), which was administered (5 mL) orally with the Suifertil pump for 18 consecutive days. The day after the last treatment (day 19), both P and M groups were randomly divided into two subgroups and gilts received 750 IU eCG i.m. (Syncrostim, Ceva Santé Animale, Libourne, France) or 300 IU FSH i.m., given in two doses 48 h apart (Gonal-f, Merck Serono, Darmstadt, Germany). In consequence, two prepubertal eCG (n = 6)- and hFSH (n = 6)-, and two mature eCG (n = 6)- and hFSH-challenged (n = 6) groups were formed. Prepubertal and mature groups were ovariectomized 72 h after eCG and 96 h after FSH administration at 200–206 days of age and 128–135 kg body weight.

Administration of one dose of eCG is enough to provoke follicle development and ovulation after about 100 h [6]. A replacement of eCG by pure FSH (r-hFSH) requires multiple injections due to FSH’s shorter half-life in blood circulation [41]. The other difficulty is the presence of the dual biological activity of eCG, provoking both FSH and LH activity in non-equid species [21]. In our preliminary experiment, we elucidated that two doses of 300 IU of commercially available r-hFSH preparation cause the occurrence of a similar number and size of preovulatory follicles 96 h after the first injection, as routinely observed for eCG given 72 h before ovary collection. Therefore, an ovariectomy performed 72 h and 96 h after eCG and h-FSH, respectively, allowed for the collection of ovaries before the ovulation of preovulatory follicles, with the preserved follicular walls and fluid needed for this study. Blood (10 mL) from all gilts was collected into ice-cold tubes containing EDTA, just before ovariectomy, by a singular semi-puncture.

#### 4.1.2. Experiment 2 [In Vitro]

Fragments of walls of preovulatory follicles from h-FSH- and eCG-primed P and M gilts were washed in a culture medium (M-199, Sigma-Aldrich, Saint Louis, MO, USA) with 10% NCS (newborn calf serum), 5% BSA (bovine serum albumin) and 100 IU/mL of penicillin, and 100 μg/mL of streptomycin. Afterwards, explants were placed in 6-well plates and pre-incubated in 1.5 mL of culture medium (supplemented with 0.1% BSA) for 2 h. Subsequently, explants were incubated with either recombinant hLH (r-hLH; 10 ng/mL, Luveris, Merck Serono, Darmstadt, Germany) or recombinant hCG (r-hCG; 10 ng/mL, Ovitrelle, Merck Serono) for 18 h at 37 °C in a humidified atmosphere containing 95% air and 5% CO_2_. After incubation, the medium with explant was placed in a clean tube and centrifuged at 1550× *g* for 10 min at 4 °C. Then, supernatant was collected and frozen at −20 °C until assayed for hormone concentrations. Each explant was weighted, snap-frozen in liquid nitrogen, and kept at −80 °C for further analysis.

#### 4.1.3. Experiment 3

To confirm that downregulation of LHCG and FSH receptors in follicular walls, triggered by gonadotropins in vitro can occur in vivo, eight mature gilts following the appearance of a minimum one estrous cycle were fed with altrenogest for 18 days. In consecutive days, animals were randomly divided into two groups and ovariectomized 72 h (approximately 50 h before ovulation; n = 4) or 120 h (approximately 5 to 10 h before expected ovulation; n = 4) after altrenogest removal. Ovary and blood collection was performed as in Experiment 1. The expected time of ovulation in gilts was estimated based on the previous data [6,42].

### 4.2. Sample Collection

Both ovaries were collected from all gilts (P, M) during ovariectomy and placed in ice-cold phosphate-buffered saline (137 mM NaCl; 27 mM KCl; 10 mM Na2HPO4; and 2 mM KH2PO4; pH 7.4) containing 100 IU of penicillin (Sigma-Aldrich) and 100 μg/mL of streptomycin (Sigma-Aldrich). Ovaries were weighed, and the size and number of follicles were evaluated on the ovarian surface (<3 mm, 3–6 mm, and >6 mm). Subsequently, follicular fluid was collected by aspiration from 4–5 healthy follicles of >4 mm diameter (defined by the limited number and diameter of preovulatory follicles in prepubertal animals; see Figure 1) and pooled, centrifuged at 1550× *g* for 10 min at 4 °C to remove cell debris, and frozen at −20 °C until assayed for hormone concentrations. Follicular walls were harvested by cutting out and peeling off the same follicle. After collection, the follicular walls were divided into 2 parts—the first part was placed in a clean tube, snap-frozen in liquid nitrogen, and kept at −80 °C for further analysis, and the second part was used in the in vitro study.

### 4.3. Steroid Hormones, Prostaglandins and pLH Assays

Steroid hormone concentrations in follicular fluid and medium were determined using radioimmunoassay (RIA) kits: A4-RIA-CT for androstenedione (A4), E2-RIA-CT for estradiol-17-beta (E2), T-RIA-CT for testosterone (T), and PROG-RIA-CT for progesterone (P4; all from DIASource, Louvain-le-Neuve, Belgium), according to the manufacturer’s instructions. Assay sensitivity was 0.03 ng/mL for A4, 2.7 pg/mL for E2, 0.5 ng/mL for T and 0.05 ng/mL for P4, and intra-assay coefficients of variation were 5.9%, 10.4%, 6.5% and 8.3%, respectively.

Prostaglandin (PG) E2 and 13,14-dihydro-15-keto PGF2α (PGFM) concentration in follicular fluid was determined using the conventional EIA method according to Blitek et al. [43]. Anti-PGE2 antibodies and anti-PGFM (donated by Dr. W. Silvia, University of Kentucky, Lexington, KY, USA, Supplementary Table S1) developed in rabbits were used to determine PGE2 and PGFM in the follicular fluid. The sensitivity of the assay was 0.19 ng/mL for PGE2 and 25 ng/mL for PGFM. The intra-assay coefficients of variation were 9.4% for PGE2 and 12.3% for PGFM.

Porcine LH (pLH) concentration in blood plasma (Experiment 3) was estimated by RIA using pLH (AVF-11043B, National Hormone and Pituitary Programme, NIDDK, Baltimore, MD, USA) for iodination and pLH (USDA-pLH-B1, National Hormone and Pituitary Programme, NIDDK) as standard and pLH antibody Sz/2/89/396, according to the method described by Ziecik et al. [44].

### 4.4. Protein Extraction

Follicular walls from P and M gilts were homogenized by sonication (Sonopuls, Bandelin Electronic GmbH & Co. KG, Berlin, Germany) on ice in lysis buffer (50 mM Tris–HCl, pH 7.4; 150 mM NaCl; 1% Triton X-100 (*v*/*v*); 0.02% sodium azide and 1 mM/L EDTA) containing 100 mM protease inhibitor cocktail (Sigma-Aldrich). The homogenates were then centrifuged at 800× *g* for 10 min at 4 °C and supernatants were stored at −80 °C until analysis. Protein concentration was measured using Bradford method [45].

### 4.5. Western Blot

Equal portions of protein (25 µg) from the follicular walls were dissolved in SDS gel-loading buffer (250 mM/L Tris–HCl, pH 6.8; 10% β-mercaptoethanol; 125 mM SDS; 40% glycerol; and 0.578 mM bromophenol blue), denatured at 95 °C for 4 min and separated on a TGX Stain-Free 10% gel (Bio-Rad, Hercules, CA, USA) at 48 mA for 1.5 h. Prior to the transfer of protein to a polyvinylidene difluoride (PVDF) membrane (Sigma-Aldrich), the TGX gel was activated to obtain the total content of loaded protein, according to the manufacturer’s instructions. Blotted membrane was blocked in 5% nonfat dried milk in TBS buffer (100 mM Tris-HCl, pH 7.4; 150 mM NaCl) containing 0.1% Tween-20 (TBS-T buffer) for 1.5 h at room temperature. Next, membrane was immunoblotted overnight at 4 °C with polyclonal rabbit or mouse antibodies: anti-STAR, anti-HSD3B1, anti-CYP17A1, anti-CYP19A1, anti-PTGFS, anti-FSHR, and anti-LHCGR (donated by Dr. Marco Bonomi, Cusano Milanino MI, Italy [46]) diluted in TBS-T buffer (Supplementary Table S1). Subsequently, membranes were washed three times in TBS-T and incubated with anti-rabbit or anti-mouse secondary antibodies conjugated with horseradish peroxidase (Bio-Rad, Supplementary Table S1) diluted in TBS-T for 1.5 h at room temperature. Afterward, membranes were washed three times in TBS-T. Immune complexes were visualized using Clarity ECL substrate (Bio-Rad) according to the manufacturer’s protocol and developed in the ChemiDoc™ Touch Imaging System (Bio-Rad). The optical density of the protein bands detected on membranes, and the intensity of the protein bands on the TGX Stain-Free gels were analyzed using Image Lab 6 software (Bio-Rad). The abundance of tested proteins was quantified and normalized to the total protein content in each equivalent lane.

### 4.6. Total RNA Isolation and Real-Time PCR

RNA isolation and gene abundance analyses were performed as previously described [14]. Briefly, total RNA was isolated from the walls of preovulatory follicles using a mirVana microRNA Isolation Kit (Invitrogen, Thermo Fisher Scientific, Waltham, MA, USA) and genomic DNA was removed by DNAse I (Invitrogen) according to the manufacturer’s instructions. RNA was quantified using a NanoDrop 1000 spectrophotometer (Thermo Fisher Scientific) and RNA quality was verified using Agilent Bioanalyzer 2100 (Agilent Technologies, Santa Clara, CA, USA). Subsequently, reverse transcription and PCR reactions were performed on 7900 HT Real-Time PCR System (Applied Biosystems, Thermo Fisher Scientific) using the TaqMan RNA-to-Ct1-Step Kit (Applied Biosystems, Thermo Fisher Scientific) and TaqMan Gene Expression Assay (20×; Supplementary Table S2). The real-time PCR Miner Software [47] was used to estimate the mean PCR amplification efficiency and cycle threshold (Ct) values for each gene. The NormFinder algorithm [48] was used to select the most stable reference among three tested genes: beta-actin (*ACTB*) and glyceraldehyde 3-phosphate dehydrogenase (*GAPDH*).

### 4.7. Statistical Analysis

Statistica 13 (Krakow, Poland) was used to perform the statistical analysis of (1) the content of steroid hormones in the follicular fluid and medium; (2) changes of mRNA abundance in the walls of preovulatory follicles; (3) changes of protein levels in the walls of preovulatory follicles. These data were compared using Student’s *t*-test. All numerical data were expressed as mean ± standard error of the mean (SEM), and differences were considered statistically significant at *p* < 0.05. In the in vitro study, one-way ANOVA with Tukey’s post-hoc test was performed to compare the control, r-hLH- and r-hCG-treated preovulatory wall explants.

## Data Availability

None of the data were deposited in an official repository. Data are available upon request from the corresponding authors.

## Supplementary Materials

The following supporting information can be downloaded at: https://www.mdpi.com/article/10.3390/ijms23169190/s1.
