# Supplementary material for: Attainment of Sexual Maturity and Gonadotropin Priming in Gilts Determine Follicular Development, Endocrine Milieu and Response to Ovulatory Triggers
Source: Int J Mol Sci. 2022 Aug 16;23(16):9190. doi: 10.3390/ijms23169190 (PMC9409084; doi:10.3390/ijms23169190)
Supplement: Supplementary file 1 [file ijms-23-09190-s001.zip › ijms-1839182-supplementary.pdf]

Supplementary Tables and Figures

Supplementary Table S1. List of antibodies used in study.

Peptide/Protein Target	Name of Antibody	Catalog No., Name of Source	Species Raised in Monoclonal or Polyclonal	Dilution used
**Prostaglandin assays**				
PGE_2	Anti-Prostaglandin E_2 Antibody	P5164 Sigma-Aldrich	Rabbit, polyclonal	1:200
PGFM	Anti-PGFM serum	WS4468-7 donated by Dr William Silvia	Rabbit, polyclonal	1:10000
**Western blot**				
CYP17A1	Anti- CYP17A1 Antibody	ab125022 Abcam	Rabbit, monoclonal	1:1000
CYP19A1	Anti-CYP19A1 Antibody	MCA2077S Bio-Rad	Mouse, monoclonal	1:250
FSHR	Anti-FSHR Antibody	bs 0895R Biossua	Rabbit, polyclonal	1:400
HSD3B1	Anti- HSD3B1 Antibody	ab55268 Abcam	Mouse, monoclonal	1:100
LHCGR	Anti- LHCGR Antibody	AD2716317 donated by Dr Marco Banoni	Mouse, monoclonal	1:600
PTGFS	Anti- PTGFS Antibody	AV48180 Sigma	Rabbit, polyclonal	1:200
STAR	Anti-StAR Antibody	ab96637 Abcam	Rabbit, polyclonal	1:400
GAPDH	Anti- GAPDH Antibody	MA5-15738 Thermo-Fisher Scientific	Mouse, monoclonal	1:2000
Anti-rabbit, secondary antibodies	Immun-Star Goat Anti-Rabbit (GAR)-HRP Conjugate	1705046 Bio-Rad	Goat, polyclonal	1:20000
Anti-mouse, secondary antibodies	Immun-Star Goat Anti-Mouse (GAM)-HRP Conjugate	1705047 Bio-Rad	Goat, polyclonal	1:20000

Supplementary Table S2. List of genes assayed in real-time PCR.

Gene symbol	Gene name	Accession number	TaqMan Assays ID	Product length (bp)
STAR	Steroidogenic Acute Regulatory Protein	NM_213755.2	Ss03381250_u1	73
HSD3B1	Hydroxy-delta-5-steroid Dehydrogenase, 3 Beta- and Steroid Delta-isomerase 1	NM_001004049.2	Ss03391752_m1	72
CYP17A1	Cytochrome P450 Family 17 Subfamily A Member 1	NM_214428.1	Ss03394945_m1	61
CYP19A1	Cytochrome P450 Family 19 Subfamily A Member 1	NM_214429.1	Ss03384876_u1	72
PTGES	Prostaglandin E Synthase	NM_001038631.1	Ss03392129_m1	70
LHCGR	Luteinizing Hormone/Choriogonadotropin Receptor	NM_214449.1	Ss03384991_u1	64
FSHR	Follicle Stimulating Hormone Receptor	NM_214386.3	Ss03384581_u1	99
*ACTB**	Beta-Actin	XM_003357928.4	Ss03376081_u1	77
*GAPDH**	Glyceraldehyde 3-phosphate Dehydrogenase	NM_001206359.1	Ss03375435_u1	75
*HPRT1**	Hypoxanthine–guanine Phosphoribosyltransferase	NM_001032376.2	Ss03388274_m1	73

*Reference genes

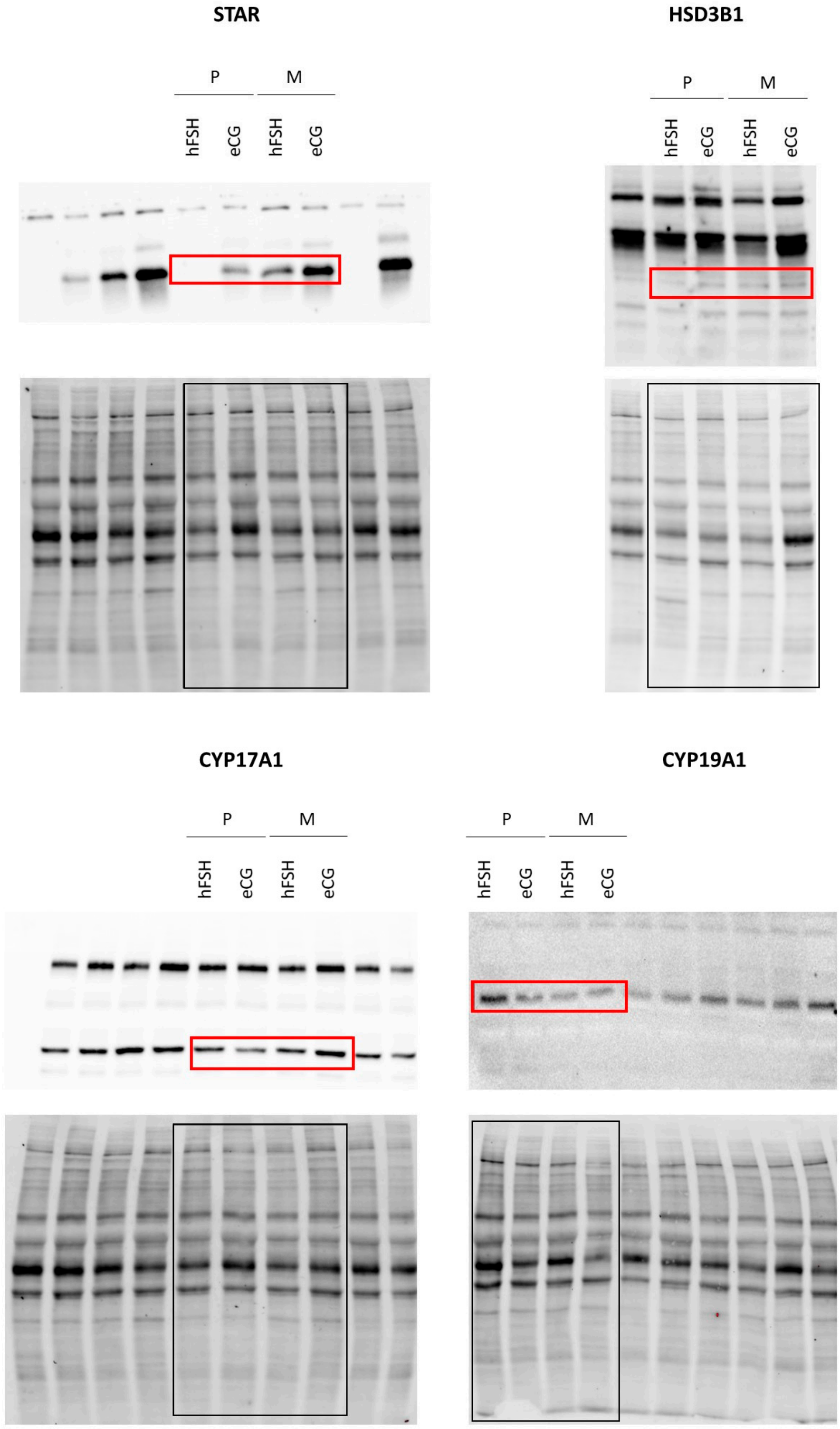

Supplementary Figure S1. Uncropped blots for STAR, HSD3B1, CYP17A1, and CYP19A1 proteins in follicles collected from prepubertal and mature gilts challenged with hFSH or eCG. In each upper panel, full blot showing STAR, HSD3B1, CYP17A1, and CYP19A1 protein expression are showed. Lower panels represent equivalent TGX Stain-Free gel showing total protein. Red boxes indicate areas presented in Figure 3. P – prepubertal, M – mature.

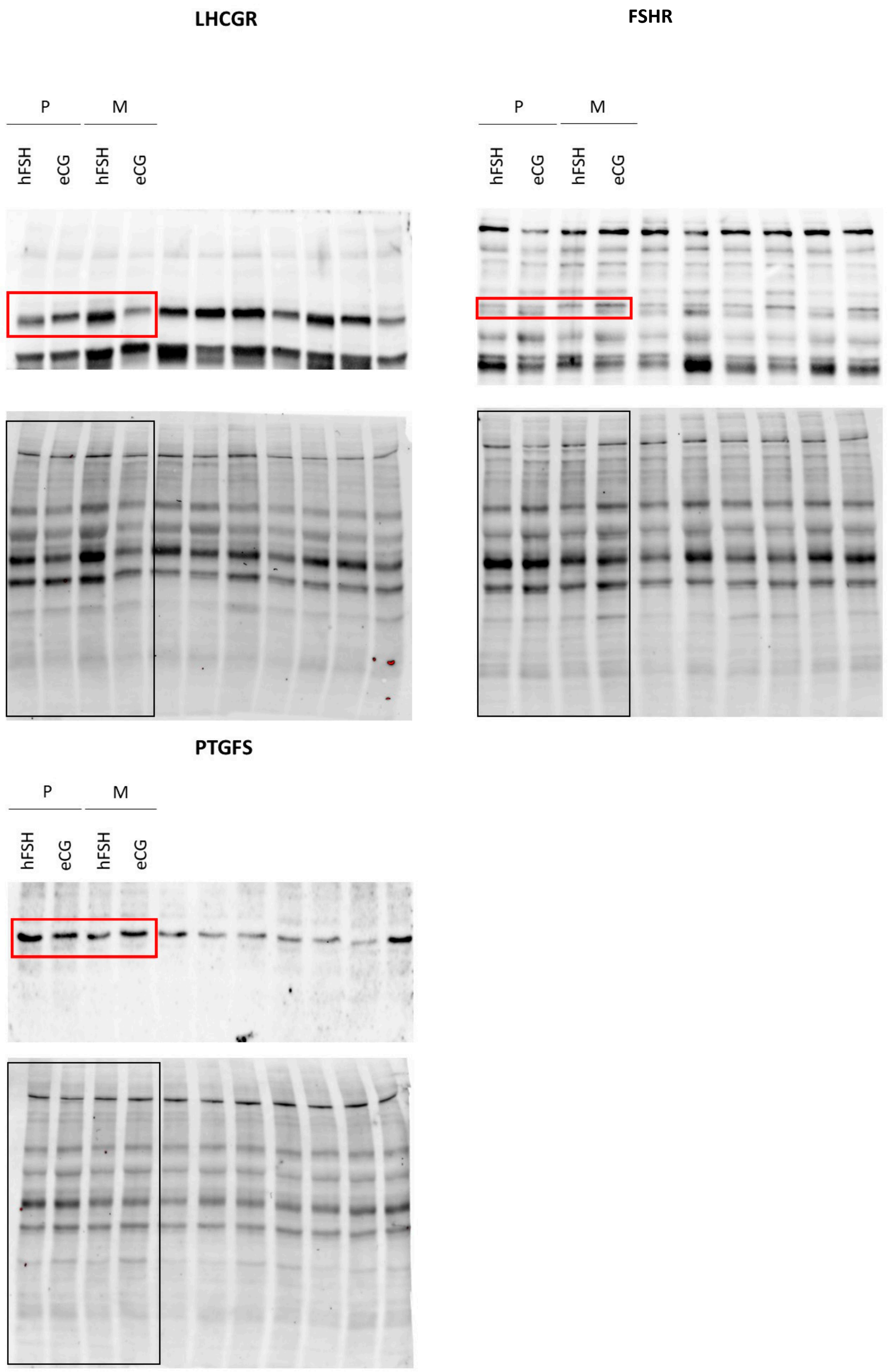

Supplementary Figure S2. Uncropped blots for LHCGR, FSHR, and PTGFS proteins in follicles collected from prepubertal and mature gilts challenged with hFSH or eCG. In each upper panel, full blot showing LHCGR, FSHR, and PTGFS protein expression are showed. Lower panels represent equivalent TGX Stain-Free gel showing total protein. Red boxes indicate areas presented in Figure 4. P – prepubertal, M – mature.

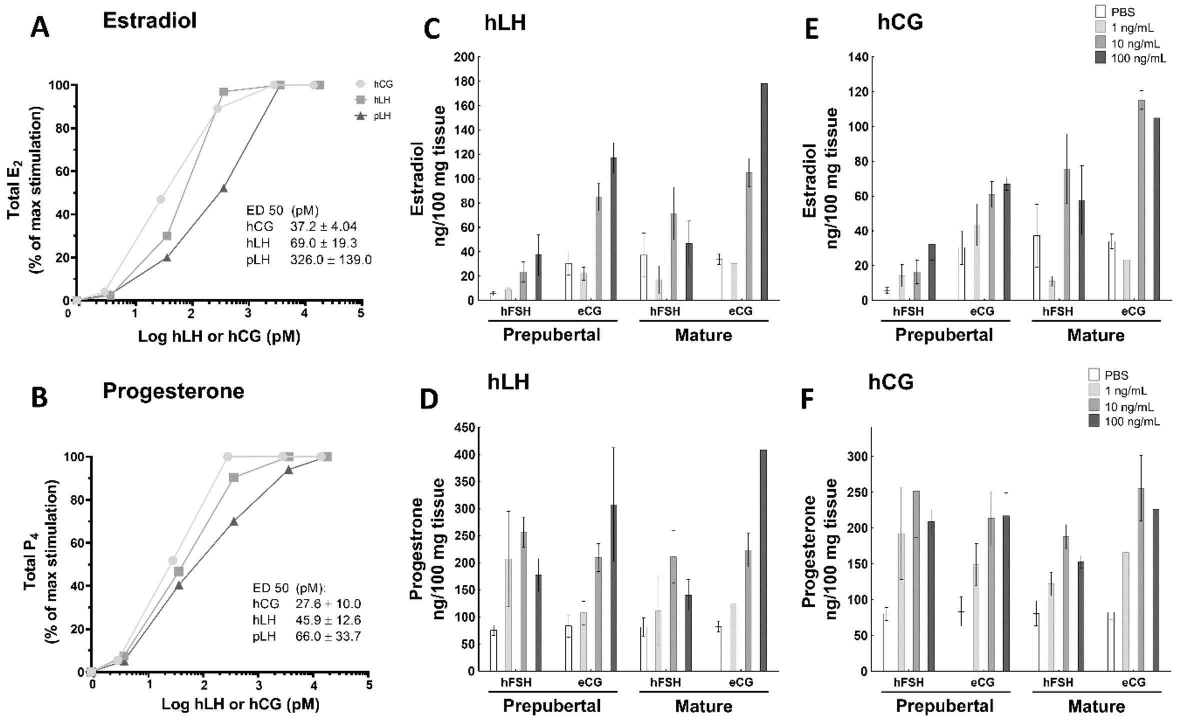

Supplementary Figure S3. Dose-response experiment. Stimulation of total E_2 (A) and P_4 (B) production by hLH and hCG. Preovulatory follicles were stimulated by increasing doses of hCG and LH. E_2 and P_4 levels were normalized as percentage of the maximal response. All the results are represented as Means ± SEM in a logarithmic X-axis, then non-linear regressions were plotted. Concentrations of E_2 (C, E) and P_4 (D, F) release in preovulatory follicles walls explants exposed to r-hLH or r-hCG (1 ng/mL, 10 ng/mL and 100 ng/mL) collected from prepubertal and mature gilts challenged with hFSH or eCG.
